# Long-term ice phenology records spanning up to 578 years for 78 lakes around the Northern Hemisphere

**DOI:** 10.1038/s41597-022-01391-6

**Published:** 2022-06-16

**Authors:** Sapna Sharma, Alessandro Filazzola, Thi Nguyen, M. Arshad Imrit, Kevin Blagrave, Damien Bouffard, Julia Daly, Harley Feldman, Natalie Feldsine, Harrie-Jan Hendricks-Franssen, Nikolay Granin, Richard Hecock, Jan Henning L’Abée-Lund, Ed Hopkins, Neil Howk, Michael Iacono, Lesley B. Knoll, Johanna Korhonen, Hilmar J. Malmquist, Włodzimierz Marszelewski, Shin-Ichiro S. Matsuzaki, Yuichi Miyabara, Kiyoshi Miyasaka, Alexander Mills, Lolita Olson, Theodore W. Peters, David C. Richardson, Dale M. Robertson, Lars Rudstam, Danielle Wain, Holly Waterfield, Gesa A. Weyhenmeyer, Brendan Wiltse, Huaxia Yao, Andry Zhdanov, John J. Magnuson

**Affiliations:** 1 Department of Biology, York University, 4700 Keele Street, Toronto, Ontario M3J 1P3 Canada; 2 Eawag, Swiss Federal Institute of Aquatic Science and Technology, Surface Waters - Research and Management, Kastanienbaum, 6047 Switzerland; 3 Department of Geology, University of Maine- Farmington, 173 High Street, Farmington, Maine 04938 USA; 4 Chanhassen, MN 55317 USA; 5 Mohonk Preserve, New Paltz, NY 12561 USA; 6 Agrosphere (IBG-3), Forschungszentrum Julich GmbH, Julich, Germany; 7 Department of Hydrology and Hydrophysics, Limnological Institute, Siberian Branch of Russian Academy of Sciences, Irkutsk, Russia; 8 Lake Detroiters Association, Detroit Lakes, MN 56501 USA; 9 Norwegian Water Resources and Energy Directorate, Box 5091, Majorstuen, N-0301 Oslo Norway; 10 Wisconsin State Climatology Office, University of Wisconsin-Madison, Madison, Wisconsin USA; 11 Bayfield, WI 54814 USA; 12 Blue Hill Observatory Science Center, PO Box 187, Readville, MA 02137 USA; 13 Itasca Biological Station and Laboratories, University of Minnesota Twin Cities, 28131 University Circle, Lake Itasca, Minnesota 56470 USA; 14 Finnish Environment Institute SYKE, Freshwater Centre, Latokartanonkaari 11, 00790 Helsinki, Finland; 15 Icelandic Museum of Natural History, Suðurlandsbraut 24, 108 Reykjavík, Iceland; 16 Nicolaus Copernicus University in Toruń, Faculty of Earth Sciences and Spatial Management, Lwowska 1, 87-100 Toruń, Poland; 17 Biodiversity Division, National Institute for Environmental Studies, Onogawa 16-2, Tsukuba, Ibaraki 305-8506 Japan; 18 Shinshu University, Kogandori 5-2-4, Suwa, Nagano, 392-0027 Japan; 19 Yatsurugi Shrine, Suwa, Nagano, 392-0024 Japan; 20 Washburn County, County Clerk’s Office, 10 4th Avenue, PO Box 639, Shell Lake, WI 54871 USA; 21 Geneva Lake Environmental Agency, George Williams College, 350 Constance Blvd., Williams Bay, WI 53191 USA; 22 Biology Department, State University of New York at New Paltz, 1 Hawk Drive, New Paltz, NY 12561 USA; 23 U.S. Geological Survey, Upper Midwest Water Science Center, Madison, WI 53726 USA; 24 Cornell Biological Field Station, Department of Natural Resources and the Environment, Cornell University, Bridgeport, NY 13030 USA; 25 7 Lakes Alliance, 137 Main Street, Belgrade Lakes, ME 04918 USA; 26 Biological Field Station, State University of New York College at Oneonta, 5838 State Hwy 80, Cooperstown, NY 13320 USA; 27 Dept. of Ecology and Genetics/Limnology, Uppsala University, 75236 Uppsala, Sweden; 28 Adirondack Watershed Institute, Paul Smith’s College, 7777 NY-30, Paul Smiths, NY 12970 USA; 29 Dorset Environmental Science Centre, Ontario Ministry of Environment, Conservation and Parks, 1026 Bellwood Acres Road, Dorset, Ontario P0A1E0 Canada; 30 Center for Limnology, University of Wisconsin-Madison, 680 North Park Street, Madison, Wisconsin 53706 USA; 31 Present Address: Basaltveien 39, N-1359 Eiksmarka, Norway

**Keywords:** Limnology, Cryospheric science

## Abstract

In recent decades, lakes have experienced unprecedented ice loss with widespread ramifications for winter ecological processes. The rapid loss of ice, resurgence of winter biology, and proliferation of remote sensing technologies, presents a unique opportunity to integrate disciplines to further understand the broad spatial and temporal patterns in ice loss and its consequences. Here, we summarize ice phenology records for 78 lakes in 12 countries across North America, Europe, and Asia to permit the inclusion and harmonization of *in situ* ice phenology observations in future interdisciplinary studies. These ice records represent some of the longest climate observations directly collected by people. We highlight the importance of applying the same definition of ice-on and ice-off within a lake across the time-series, regardless of how the ice is observed, to broaden our understanding of ice loss across vast spatial and temporal scales.

## Abstract (summary)

**Table:** 

Measurement(s)	Ice-on and ice-off dates • Lake characteristics
Technology Type(s)	Observations • Historical records
Sample Characteristic - Environment	ice-covered lake
Sample Characteristic - Location	Northern Hemisphere

## Background & Summary

People have been recording the dates lakes freeze and thaw every winter from decades to centuries across hundreds of lakes because of the importance of ice to refrigeration, hydropower generation, winter transportation, and recreation^1^. For example, one of the oldest ice and climate datasets directly collected by people was started in 1443 for Lake Suwa, Japan by Shinto priests because of the importance of ice formation to their Shinto traditions^2^. Moreover, long ice records increase our understanding of climatic changes since the Industrial Revolution as ice phenology (the timing of ice-on and ice-off) is a sensitive indicator of climate^2–4^.

In 1996, a network of lake ice researchers and their data from around the Northern Hemisphere was formed, called the Lake Ice Analysis Group (LIAG) led by John Magnuson from the University of Wisconsin-Madison^5,6^. The purpose of LIAG was to form a network of researchers so that they could discover patterns not visible in short time series as well as uncover similarities and differences at broader spatial scales than previously analyzed, such as a lake, province, state, or country. They used the ideas of “Invisible present”^7^ and the “Invisible place”^8^ to make clear the importance of expanding the time and the space scales of ecological research to understand the world around us^6^. The first LIAG group from North America, Europe, and Asia met at the Center for Limnology’s Trout Lake Station in Wisconsin, United States, to share the data amongst the group and more generally at the U.S. National Oceanic and Atmospheric Agency’s National Snow and Ice Data Center. The initial data set was developed by John Magnuson and Barbara Benson from the Center for Limnology, University of Wisconsin-Madison and Dale Robertson with the United States Geological Survey resulting in the first Hemisphere-wide comparisons^4^. The next major effort to update the data and expand the number of lakes was led by John Magnuson and Barbara Benson^9^. A new and expanded lake ice network with some old members and many new contributors has since been developed and led by Sapna Sharma^10,11^ to update and extend the lake ice seasonality data set; the present paper is part of that effort^6^. The ice seasonality data continue to be archived at the National Snow and Ice Data Center. Climate change was apparent in the earliest LIAG papers^12^ and trends in climate warming appear to be accelerating in recent decades. Today the United States Environmental Protection Agency considers changes in lake ice seasonality as one of the strong indicators of climate change (https://www.epa.gov/climate-indicators).

In recent decades, the proliferation and advances in remote sensing technologies are enabling the collection of ice records from many regions of the world where *in situ* observations do not exist or have not been updated^13^. Rapid losses in seasonal ice cover in recent decades^10,14^ highlight the urgency of integrating *in situ* and remote observations to obtain both a broad spatial and temporal understanding of ice loss, which is currently not possible with either of these approaches independently. However, the harmonization of data from both disciplines requires well-documented definitions and the historical context of the recorded observational data. Here, for the first time, we provide detailed records of *in situ* ice observations for some of the longest studied lakes, in addition to lakes from countries that have not been previously included in global synthesis studies^4,10^.

Across lakes, a standard definition for ice phenology does not yet exist. For example, the timing of ice-on and ice-off data may be defined either based on a percentage of ice coverage or the ability of ships and boats to navigate across the lake^13,15^. Regardless, the same definition for ice-on and ice-off should be applied for a lake over its time series to limit the uncertainty within the ice record and to assess long-term trends and patterns in ice phenology^4^. In some cases, lakes may have inconsistent standards of judging ice phenology information over time, and this is especially concerning when integrating *in situ* and remote sensing observations. For some lakes, additional information on freeze/thaw events within a season, ice thickness, and ice quality, may exist and are useful in understanding ecological under-ice dynamics^13,16,17^, such as water temperatures^18^, primary production^16^, and fish biodiversity^19^.

Here, we provide ice phenology records and their corresponding meta-data for 78 lakes from 12 countries in North America, Europe, and Asia. These lakes comprise some of the longest ice phenology records collected around the Northern Hemisphere (>100 years) and also include lakes from countries such as Canada, Iceland, Norway, and Poland, that have typically not been included in global synthesis studies or within the National Snow and Ice Data Centre (NSIDC) Lake and River Ice Phenology Dataset (LIAG^20^). In this study, in addition to the ice phenological records, we asked: i) What are the physical characteristics of the lakes?; ii) What were the definitions of ice-on and ice-off?; iii) How were ice observations made and recorded?; iv) Why was the ice record started historically?; and vi) Did the methodology change over the entire time series?

## Methods

**Fig. 1:**
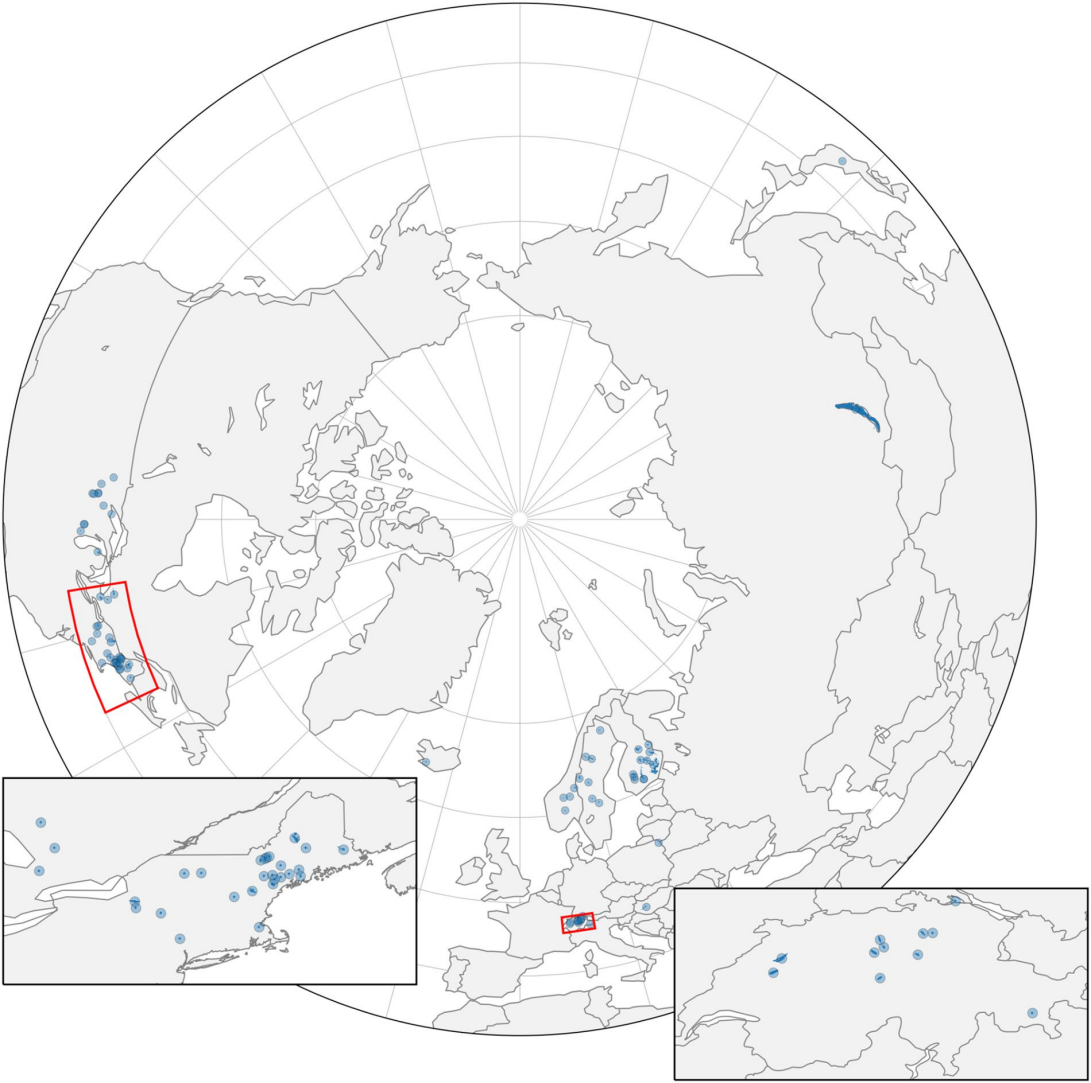
Lakes with ice phenology data described in this dataset are outlined and filled in blue, using HydroLAKES polygons (Messager *et al*.^44^). A blue highlight is added to better identify the locations of even the smallest lakes. Insets show detail of the northeastern U.S. and Swiss lakes’ positions.

We compiled *in situ* ice phenology records and corresponding physical characteristics for 78 lakes spanning up to 578 years long (Fig. 1). Below we provide the sampling methodology and the definitions of ice-on and ice-off for each of the lakes provided by data contributors and organized by geographic region. Varying amounts of information were available for each lake.

### North America

We summarize ice phenology records for the lakes by province in Canada and state in the United States of America.

### Ontario, Canada

The ice record of *Lake of Bays* was started in 1908 by Brian Tapley’s family for fishing and boating purposes. Visual observations to identify when open water was observed across most of the lake were consistently made by the Tapley family over many generations. They conducted their observations from the shoreline of a large bay area facing towards the lake during the daytime. Although data are subject to personal judgment of untrained observers, the uncertainty remained low since the observations were done by the same family in the same manner each year.

*Lake Nipissing*’s earliest ice record goes back to 1901 and reported annually by the North Bay Nugget Newspaper for boating, fishing, and recreation purposes. Visual observations to identify when open water was seen across most of the lake were made by local residents, and subsequently collected and summarized by the North Bay Nugget Newspaper. Observers stood on the shoreline of the larger bay areas and faced toward the lake. The procedures for recording and collecting data were considered to be consistent during the whole period.

*Lake Simcoe* had its ice-on data first reported in Kempenfelt Bay in 1852. The earliest ice records were believed to be provided by the local newspapers for cultural and economic interests to Barrie citizens such as the February carnival, ice fishing, and ice harvesting. Ice phenology records were collected solely via visual observations. Observers would stand on the municipality of Barrie shoreline at the west end of Kempenfelt Bay and look due east for ice coverage information to identify when the west end of Kempenfelt Bay was completely frozen in the winter or ice-free in the spring. Remote sensing images from the Moderate Resolution Imaging Spectroradiometer (MODIS) were recently incorporated to supplement two recent missing years in the spring of 2004 and fall of 2017. There is a limited uncertainty because of subjectivity of observers, inconsistent numbers of observers and observing locations during the data collecting period, and possible historical errors associated with the early start of the dataset in 1852.

### Maine, United States

Ice-out data for New England, including Maine, New Hampshire, and Massachusetts, were compiled and reported by Hodgkins and James^21^ then updated in Hodgkins^22^. These comprehensive compilations systematically identified known observers, data sources, and some site-specific notes on methodology through 2008, relying substantially on an earlier compilation by C.B. Fobes^23^. As noted by the authors, original observation methodology was frequently not recorded or reported, and ice-off is generally defined as the date when winter ice disappears from the lake^21^. A more in-depth discussion of shoreline morphology and variability in reported ice-out dates in Hodgkins *et al*.^24^, states that the shape of some of the lakes in this compilation show increased variability between reporters if the lake had one particularly long axis. Ice on these long, narrow lakes might clear at one end but not the other, yielding a difference in reported ice out of several days while relatively round lakes had much smaller variability^21^.

*Lake Auburn*’s ice phenology record began in 1863. The local power company and Auburn water district started the ice record and publicly shared ice-off dates when the winter ice disappears in the spring in local newspapers. All of the information was based on visual observations of multiple observers including Erica Kidd of Auburn Water District who reported the date in 2020. A comparison between two data sources (the state Bureau of Parks and Lands and USGS) with 5 overlapping years differ by +/− 1 day in most years.

*China Lake*’s ice record began in 1874 by the China Lake Association based on visual observations of Kennebec Water District staff. Dates were initially recorded for most years 1874–1883 then not recorded consistently until 1932^21^. The lake ice off data is recorded when the lake is mostly free of visible ice and is navigable by boat. Observations were conducted eastward between 6:30 am and 3:00 pm at the East Vassalboro boat launch.

*Cobbosseecontee Lake*’s ice phenology record began in 1840. The record started in 1840 with Sara Farr, Mary Oatway, Bill Schenck, and Nancy Schenck noted as visual observers of the date that lake ice completely disappears in the spring^22^. In 1974 and 1994, data were also recorded by a secondary observer. These ice-off dates were adjusted by the average difference between the primary and secondary observers.

*Damariscotta Lake*’s ice observations were first recorded in 1837. Ice-off for most years was observed from the northern end of the lake^21^. The record was started based on visual observations made by three generations of Fred Jackson’s family, James Birkett, and the Maine Bureau of Parks and Lands^21^. A secondary observer recorded dates for 1959, 1962–68, 1970–78, and 1997–98; for these years, ice-off dates were adjusted by the average difference in the dates between the primary and secondary observers^21^.

*Kezar Lake*’s earliest ice phenology record is 1901 by Arthur P. Stone (1901–1961) and kept at the Lovell town office. There are no missing years in the time series. According to the current observers, John Bacchiochi and Stan Tupaj, who have been conducting observations for about the past two decades, ice-off date is defined as the date when navigation is possible from one end to the other end of the lake. This also means that there might be ice in the coves and some other areas within the lake. Since Kezar Lake is 14.5 kilometres long and the Lower Bay is relatively shallow (~4.5 m) compared to the rest of the lake, the Lower Bay opens much earlier. Observations are made above the Lower Bay where observers look at the lake from both the eastern and western shores, as well as the North End. Each year, data are collected from various vantage points to obtain a more complete view of Kezar Lake. The same observation method has been applied since ~2000 in order to maintain consistency in the data. However the residents who recorded the ice phenology information before John Bacchiochi and Stan Tupaj (1962 - ~1999), would collect data from the north end of the lake where, at ~48 metres, it is the deepest part of the lake, when they could access different observation locations. For example, the data collected from different sources for overlapping years (2008–2018) show agreement between different observers in some years, and up to two days of disagreement for other years.

*Moosehead Lake*’s ice phenology record extends back to 1848. Ice-off date is defined as “the earliest date of open navigation from Greenville to Northeast Carry^21^”. The ice-off record was started by observers for the Kennebec Water Power Company and continued by the Maine Department of Inland Fisheries and Wildlife using visual observations^21^. Recently, small aircraft have also been collecting ice-off data and the current observer is pilot Roger Currier.

*Pennesseewassee (Norway) Lake*’s ice record began in 1874 by Woodman’s Sporting Goods, located near the southern end of the lake. Multiple observers have recorded ice data when annual winter ice cover disappears from the lake with the most recent reporter being the current lake association president, Sal Girifalco. Two residents on the lake are asked to report ice-out dates each year to make sure there is no bias because of observation location.

*Rangeley Lake*’s earliest ice phenology information was started by employees of the Union Water Power Company in 1880 and continues to present-day each year based on visual observations of the date when ice completely disappears from the lake. More recent dates have been reported by employees for FPL (Florida Power & Light) Energy^22^.

*Richardson Lake*’s ice record began in 1880 and *Mooselookmeguntic Lake*’s ice record began in 1884 by the Union Water Power company; these two lakes are located quite near each other and have similar ice-off reporting histories. The records are associated with data collection on water level management for electricity generation on dams. Ice-off dates are recorded when winter ice previously covering the lake disappears in spring^21^. Different observers are listed in both records.

*Sebago Lake*’s ice record began in 1870 with the earliest reporter believed to be the Maine Sunday Telegram. Ice-off dates were recorded by the owners of Jordan’s Store in the town of Sebago who note the disappearance of the ice across the Big Bay on the west shore of the lake. If Big Bay does not completely freeze over, the data is recorded as “No complete ice cover” and assigned a day of year of 70 in the original data files. A Standard Operating Procedure (SOP) has been established by observers through the ice record, primarily the proprietor of Jordan’s Store in the town of Sebago, as well as Brie Holme of the Portland Water District, who reports the date.

*Sebec Lake*’s ice record was started in 1897 by Bill Elliott^22^. The first day, when navigation from one end to another end of the lake is possible, is recorded as the lake ice-off date^22^. In the past, data were collected via visual observations. More recent information may be additionally gathered from webcam images along the lake.

*Thompson Lake*’s ice record was started by Joseph Trebilcook in 1902^22^. Since then, several different observers have been recording ice-off dates including Kathryn Cain and the Thompson Lake Environmental Association who are currently collecting these data. Ice-off date is recorded as the date a watercraft can navigate up and down the lake (north and south) without obstruction of ice sheets. However, ice may remain in the coves.

*West Grand Lake*’s ice phenology record began in 1878 by Marion Staples’ family. Marion Staples’ grandmother started the record to inform sport fishermen at the family sporting camp. Ice-off dates are generally defined as the date when the annual winter ice previously covering the lake disappears in spring^22^.

*Wilson Lake*’s ice record started in 1889. Several data collectors have been providing information throughout the time series, including Bruce Dyke (Wilton Fish & Game Association) and Robert Lively (Friends of Wilson Lake) who are currently recording ice-off dates. Ice-off is defined as when a boat can travel from the boat launch to the big island (southeast to northwest across lake). Different observers sometimes report dates that are one day apart for the same year.

### Massachusetts, United States

The ice record in *Houghton’s Pond*, also called *Lake Hoosic-Whisick*, began in 1886 by the staff of Blue Hill Observatory who have been collecting weather and climate data daily for well over a century (*Graphs of Annual Blue Hill Observatory Climate Data*) based on visual observations. Ice cover is observed daily from the summit (where the observatory is located 145 m above the pond) with the assistance of binoculars if necessary. When the pond is not visible from the summit, observations are made at the shoreline. The pond is considered thawed when a trace amount (five percent or less) of ice remains after the last full freeze. Ice-on is also recorded at this site as the first date with complete ice coverage; intermittent ice coverage is tracked by the observers.

### Michigan, United States

*Grand Traverse Bay* in Lake Michigan ice record started in 1850 which along with Bayfield Bay in Lake Superior^15^ is the longest ice phenology dataset for the Great Lakes. The ice-on date is recorded when the lake is covered with ice over West Bay from the southern shore to Power Island^25^. Data were collected to provide information for transportation, fishing, hunting during the winters and more recently to monitor climate change over the past several centuries^25^.

### Minnesota, United States

*Christmas Lake*’s ice record started in 1887. For the past 22 years, Harley Feldman has visually observed ice cover several times per day from fall to spring facing south on his house deck. Before 1998, the data was documented in the Lake Association paper records and has been passed down from previous Christmas Lake Homeowners Association secretaries and treasurers. Ice-off date is defined as when there is no permanent visible ice on the lake in the spring. If the lake re-freezes, the ice-off date is moved out until the next date when there is no visible ice on the lake.

*Clear Lake*’s ice record which is composed of ice-on and ice-off dates was started in 1874. Over the time series, records have been kept by local residents and newspapers including (i) Waseca Paper “Minnesota Radical” in 1874–1880; (ii) Mrs Orrin Smith and Mrs Herb Starr in 1894–1939; (iii) Marvin Johnson in 1940–1979; (iv) Edgar Johnson in 1980–1988, (v) Richard and Phyllis Fisher in 1990–2001, (vi) Don and Bob Lynch, Ron Fuchs, Dean Hagen, and Bill Severin in 1987–2008; (vii) University of MN SROC and the Waseca Lakes Association since 2001 until present. The ice-on date of the lake is defined as the first day in the winter season when the lake is completely frozen without temporal thaw. The ice-off date is defined as the day when 90% of the ice is melted. Data have been collected based on visual observations at multiple sites and directions as it is easy to drive around the perimeter of the lake.

The ice record for *Detroit Lake* was started in 1893 for ice-off and 1910 for ice-on. The earliest data were collected by unaided visual observations, while after 1993, data have been collected through a variety of methodologies including telescope, drive-by, and consultation with Dick Hecock. As Hecock resides on the north shore of Big Detroit near the sandbar dividing the lake into Big and Little Detroit bays, he can conduct observations of a large portion of Little Detroit and most of Big Detroit with a telescope or in close distance if the viewing weather is poor. Secondary observations for ice-out dates are also collected from two other residents on Big Detroit south shore. More daily observations, many of which are in the early morning, are made when the ice-off day is approaching. Ice-on date of Detroit Lake is defined as the date when the lake is frozen without substantial further openings because of thaws according to the MPCA (Minnesota Pollution Control Agency). Ice-off date is defined as the date the entire lake is “essentially ice-free”. The southwest shore is monitored more carefully since it is almost always the last portion of the lake to thaw. Hecock has been following guidance established by the MPCA since he took over the record in 1993. However, the uncertainty of the earlier data was in the order of about five days because of the lack of knowledge of former observers, inconsistency in data definitions, and an incomplete view of the lake. Before roads were built around Detroit Lake, the lake area that froze last was furthest away from the town where early observations were made so that ice coverage might have been overestimated. The ice-off date might also have been recorded earlier than the actual date because Little Detroit, which was closer to the town, typically thawed earlier than the deeper parts of Big Detroit found along the south shore of the lake.

*Lake Minnetonka*’s ice phenology record started in 1855. Observers, who are selected members of the Freshwater Society Board or Staff, along with deputies from the Hennepin County Sheriff’s Water Patrol, collect data in the watercraft three times per day including at night when ice-off date approaches. After 2014, web cameras located at certain yacht clubs and marinas were used to supplement visual ice observations. The ice-off date is currently defined as when a watercraft is able to travel from any shore to any other shore without being stopped by hard ice. Floating ice is allowed if the watercraft can drive through it. In some of the previous years, climatologists or weather forecasters recorded ice-off dates based on different criteria from the one that is currently used.

The ice phenology record of *Shields Lake* began in 1894. Over the time series, the transfer of responsibility for recording has been passed down by different individuals living near the lake with Larry Smisek as the most current data contributor. Smisek started collecting ice phenology data in 2013 by visually observing the lake from the northeast side of the lake, which has often been the last location of the lake to thaw. Ice-off date is recorded when 95% of the lake is open water.

### New Hampshire, United States

*Lake Sunapee*’s ice record was started by the Osborne family in 1869 who have continued observing ice phenology across several generations. The ice-off dates are defined as the date it is possible to navigate on a boat from George’s Mills at the northern end of the lake to Newbury Harbor at the southern end. This path transects the long axis of the lake and is adjacent to the Lake Sunapee buoy^26^.

*Umbagog Lake*’s ice record was likely started in association with water level management for power generation in 1880 by the Union Water Power Company. Ice-off dates are generally defined as the date when the annual winter ice previously covering the lake disappears in spring^21^.

*Lake Winnipesaukee*’s earliest ice phenology data was recorded in 1887. Ice-off date is recorded as the date when all of the ports for the cruise boat Mount Washington including Alton Bay, Weirs Beach, Center Harbor, Meredith and Wolfeboro are free of ice. The record was first used to track the availability of commercial and passenger transport across the lake based on visual observations from multiple points and different directions around the lake. Modern data are collected by Dave Emerson who flies over the lake two to three times per day for ice coverage information.

### New York, United States

*Cazenovia Lake*’s ice record started in 1839. Ice-on date is defined as when the lake is covered with ice and ice-off date is defined as the date that most of the lake is open water. For Cazenovia Lake, freeze and thaw events are recorded as dates when ice breaks up and refreezes. The data on Cazenovia Lake was checked, corrected, and complemented through a review by P.G. Rudstam of available diaries at the Lorenzo Mansion State Historical Site on Cazenovia Lake, and information from Dr. Ken Stewart, University of Buffalo. The diaries were kept from 1839 by consecutive owners of the Lorenzo Mansion (members of the Ledyard family) and are kept at the library of the Lorenzo Mansion State Historical Site. Data after 2008 are from Mr. Phillip Hart, Cazenovia, and his father before him, kept track of these dates for some time and is the source of Ken Stewart’s earlier data. The original Ken Stewart dates were maintained when there was a discrepancy between records from the Hart family and Stewart's assessment. Mr. Hart states that he checks the ice by driving around the lake and that he does not count ice-on dates if the lake broke up again the same day (i.e., lake skimmed over with ice for a short time). Over the course of the time-series, changes in data collection procedures were documented in the database.

*Mirror Lake* is located in the Adirondacks with ice phenology information collected from 1903 onwards. Ice-on date is recorded when the lake is covered completely with ice. The ice-off date is the first day after winter ice cover when the lake is mostly free of ice, except possibly small pieces of ice near the shore. Freeze-thaw events, usually at the time of ice formation, are additionally noted. For example, the lake may freeze and melt a day later, then freeze for the rest of the season after two weeks. Lake Placid residents started collecting the data in 1903, most recently data collection has been in coordination with Judith Shea of the Lake Placid Volunteer Fire Department and Dr. Brendan Wiltse, Adirondack Watershed Institute Water Quality Director. Data were collected based on visual observations over the entire lake from the lake shore. Recently, data were supplemented with a 24/7 live feed from a webcam observing the main portion of the lake from the south looking north. When ice-on or ice-off dates near, the lake is further observed in person. Since the historical definition of ice-on and ice-off data have not been well documented, there may be an uncertainty corresponding to the time needed for ice-off to occur. Over the time series, there are no missing years in the ice-off dataset, but 11 years are missing from the ice-on record.

The first observation of ice was recorded from *Mohonk Lake* in 1932. Both ice-on and ice-off dates have been collected since 1932-present. The Smiley Family began recording weather data in 1896 and Daniel Smiley began recording lake ice phenology dates in 1932 each year until his passing in 1989. Daniel Smiley Research Center staff assumed all data collection after Dan passed away and in 2014, a small group of citizen science volunteers named the “Climate Trackers” was trained to collect the data which has continued through 2020, with substitutions by Research Center staff. Ice-on date is defined as the first date of the season when lake ice coverage is 100% and ice-off date is defined as the first date after ice-on when lake ice coverage is completely absent (i.e., 0%). Further, the percent coverage of lake ice is recorded consistently throughout the season. For instance, if the lake is half frozen, 50% lake ice is recorded. Observers stand at the end of the Mohonk Lake Boat Dock and look toward the southernmost end of the lake from 4 pm to 6 pm to collect information on ice coverage. Additional information, such as ice thickness, was recorded from the center of the lake when it was safe for people to cross. The procedure for collecting data has remained consistent over the time series.

*Oneida Lake*’s ice record began in 1827 with the earliest ice limnological data obtained from two diaries: (i) Asa Eastwood’s diary (1827–1865; kept at the Syracuse University Library’s rare book collection) interpreted by Jack Henke^27^ and (ii) James Bernard’s diary (1846–1901; kept by the family) interpreted by E. Mills and C. Hoffman of the Cornell Biological Field Station (CBFS). During the overlapping period of the two diaries (1846–1965), the Bernard diary data was used since that diary also included ice-on dates. The diary data replaces previous data from 1844 to 1900 that were found to be in error. Note that these problematic data were used by Magnuson *et al*.^4^, but corrected in subsequent papers from that group e.g.^28^. Data obtained from 1901 to 1974 were from seven different observers and were kept at the Oneida Fish Culture Station. After 1975, CBFS personnel have been observing ice conditions in the middle of the lake (Shackelton Point) due East, West, and North in both the mornings and afternoons. Further, the amount of open water is noted. For Oneida Lake, ice-on dates are more difficult to define as the ice usually temporarily forms and breaks up again before the final freeze over. This dataset follows the convention of the National Ice Database which states that the first date that the lake completely froze over is recorded as the ice-on date. Ice-off date is better defined as ice often leaves the lake in a few days. However, when the observations are questionable, ice-off date is defined (since 1975) as the date when it is possible to drive a boat from the south to the north shore at a mid-lake location by Shackelton Point at the CBFS. Freeze and thaw events between the first ice-on date and the ice-off date are also recorded. Ice duration is calculated from the day of first ice-on to the day of ice-off and subtracting the number of days the lake was open in between. In the winter of 2001–02, Oneida Lake did not freeze completely so there is no ice-on date. However, there was some ice on the lake and most of that ice had melted by the provided ice-off date. In 2006, half of the lake opened up on March 15th, 2006 and most ice disappeared on April 1st, 2006. For that year we used March 15th to calculate the duration of ice cover, but April 1st as the date when most ice was absent from the lake (ice-out date). When notes indicate the presence of short ice-on periods prior to the given first ice-on date, a three-day ice duration is added to those periods when calculating ice duration for that winter.

*Otsego Lake*’s ice phenology record begins in 1849. Ice thickness data exists for the winters of 1970–71 to 1974–75. The dataset is currently maintained by the SUNY Oneonta Biological Field Station. Records from 1843–1972 were kept by a series of Weather Observers, beginning with George Pomeroy Keese (1843–1910), followed by Elizabeth Cooper Keese (1910–1930), and then staff of a local newspaper the *Freeman’s Journal* (1930–1972). Those data were compiled and described in a 1973 Climatological Summary^29^. Definitions of ice-on and ice-off were described for 1843–1930: ice-on date was recorded when the lake was “entirely closed by ice”, ice-off date recorded when “the last ice left the lake”. It has not been confirmed that data collected from 1930–1967 followed the same convention or that observations were made of the entire lake, which may increase the uncertainty of ice-on dates for those years. If observations were made from the Village of Cooperstown (at the southern end of the lake), the area of the lake that usually freezes last is not visible from that location. It is possible that freeze dates prior to ~1950 reflect the condition in the southern end of the lake, and thus may report earlier freeze than would be experienced mid-lake. According to local anecdotes, large sums of money and other commodities were gambled on the ice-out date, so the accuracy of these recorded dates would have been of high importance. The records would have served to inform and document many commercial and recreational activities in Cooperstown that relied on lake ice including commercial ice fishing for lake whitefish, ice harvesting for summer use, transporting over the ice for construction, and maintenance and repair of lake-side summer cottages. In 1967, a Biological Field Station was established in Cooperstown by the State University of New York (SUNY) College at Oneonta and Otsego Lake became a focus of research efforts^30^. Ice records are maintained by this institution to the present. Ice observations are made by the faculty and staff and occasionally by trained lakeshore residents who will drive around the lake to obtain an entire view of the lake. When observers suspect the lake is ready to freeze, observations of ice conditions are prioritized. Location and size of openings are further noted, but not consistently tracked. One freeze/thaw event was recorded for the winter of 2005–6 when the lake completely thawed and then completely froze again. Total duration of ice cover has been reported as the total number of days with complete ice cover, excluding the span during the thaw.

### Vermont, United States

*Lake Champlain* is located on the border between the United States and Canada and its ice record began in 1814. For Lake Champlain, ice-on dates are referred to as “closure” dates which can be interpreted as cessation of commercial shipping traffic or full ice coverage on the lake from Burlington, Vermont directly west/northwest of Port Kent, New York. The ice-on data were collected from local newspapers and multiple observers including: (i) U.S. Weather Bureau Climatological Record Book in Burlington, Vermont, and Burlington Free Press clipping (Data sources unknown) from 1816–1871; (ii) Charles E. Allen from 1872–1886; (iii) cooperative weather observer Mr. W. B. Gates from 1886–1906; (iv) U.S. Weather Bureau and National Weather Service from 1906-present. Throughout the period, historical records from Shoreham, Vermont, and Old Fort Ticonderoga provided additional information. It is noted that small stretches, or gaps of open water may still exist on portions of the lake which are too small to be observed by the satellite data.

### Wisconsin, United States

*Green Lake*’s earliest ice records began in 1939. Ice-on dates are defined when a complete line between Sandstone Point and Robin Hood Point is frozen; therefore, ice does not necessarily need to cover the entire lake. Ice-off dates are defined as the date when it is possible for a boat to navigate from the east end to the west end of the lake. The presence of ice chunks is acceptable at this time. Freeze/thaw events within winter are occasionally recorded for Green Lake. Since Carl Mapps started the ice record in 1939, several different observers who lived on Robin Hood Point have been collecting ice phenology data with no missing years. Citizen scientists visually observe the lake from Robin Hood camp point toward Sandstone point. Observers, of which Joseph Norton of Norton Boat Works and Paulette Janssen of the Green Lake Sanitary District are the most recent, try to follow earlier protocols so that the observation methodology remains consistent throughout the time series. Recently, a secondary record with the dates of complete ice cover has been started to complement the record using the original methodology.

*Geneva Lake*’s ice record began in 1862. Theodore W. Peters has been recording the data for the last 25 years. Geneva Lake typically freezes from the east end to the west end. The deepest area of the lake is the last area to freeze. Peters observes the ice from the west end of the lake at the following points: Conference Point, Cedar Point, George Williams College, Brookwood Subdivision lakefront, Fontana, and Williams Bay lakefront while looking towards the lake and scanning for presence of ice with binoculars. Observations are sometimes made multiple times a day with the first observation at around 8 am if the conditions are appropriate. It is easier to detect open water if there is light snowfall. Ice-on date is recorded as the date the lake has been frozen for two days. However, waterfowl will often keep water open, so up to about five acres of open water full of waterfowl is acceptable for the lake to be categorized as frozen. The ice-off date is defined as when the lake is free of ice other than small ice plates that float around or are washed up on the leeward shore of the lake. Freeze/thaw events within a winter are additionally recorded in the database. Factors such as variation in observation point, weather, and ability to see last ice formation contribute to uncertainty in the recent data. The uncertainty of early data may be different because the collecting procedures may not be the same as the one currently applied. The lake has not completely frozen in five of the last 23 years.

*Lake Mendota*’s ice record began in 1852 and *Lake Monona*’s ice record began in 1851. The earliest ice information for the two large lakes that border Wisconsin’s capital city of Madison, was gathered initially from newspapers. By the 1880s, the ice records were maintained by weather observers at Washburn Observatory and the U.S. Weather Bureau Office at North Hall on the University of Wisconsin-Madison campus. For the last six decades, staff from the Wisconsin State Climatology Office have maintained the ice record, which continues with Lyle Anderson and Ed Hopkins. Because of the irregular shapes of these two large lakes, each lake cannot be viewed in their entirety from one location. Thus, observations are conducted for Lake Mendota from multiple points including Maple Bluff Beach due west direction, Tenney Park due north and west of the locks directions, and Observatory Hill due north direction. For Lake Monona, observers view the lake from: boat launch at Olin Park due east-northeast; BB Clarke Beach due south; Few Street due south; along streets along the north shore of the lake to the east of the Yahara River due south and southeast; and Esther Beach due north. Observations for both lakes are made during the day by multiple observers and cross checked with each other afterward. Webcams on the Space Science and Engineering Building roof at the University of Wisconsin-Madison are consulted during the daylight when the observers are not near the lakes. For Lake Mendota, the date of ice-on is recorded when at least 50% of the lake is frozen and the line between Picnic Point and Maple Bluff is covered with ice, a tradition that goes back for probably a century. When at least 50% of Lake Mendota is ice free and the line between Picnic Point and Maple Bluff is open, the date is recorded as the ice-off date. Ice-on date for Lake Monona is defined as the date when at least 50% of the lake is frozen. Lake Monona ice-off date is defined when 50% of the lake is open water. Multiple freeze/thaw events that occur primarily at the start of the ice season are placed into the records; ice openings or closures of less than 24 hours are not considered. Variations in data collecting procedures undoubtedly occurred in the mid-19th century, but Anderson and Hopkins have strived to reduce current discrepancies as they observe the lakes more routinely and at more different angles than in the early years. Uncertainty may remain due to visual estimation of the percentage of the lake actually covered, foggy or low visibility, and low stability of thin ice sheets that break within 24 hours.

*Shell Lake*’s ice phenology record was started in 1905 based on visual observations made by several individuals of the Washburn County Clerk’s Office, including O.J. Soholt (1931–1981), Jack Brown (1981–2006), Lynn Hoeppner (2007–2012), and Lolita Olson (2013 - present). Observations are conducted from Rolph’s Point where observers look in the northeast direction as described in the Standard Operating Procedure (SOP) which is passed among observers. The ice-on date is recorded when the lake is completely frozen, which is typically when the deepest area of the lake freezes. Ice-off date is defined as the date when the lake is completely thawed with no ice at the deepest area of the lake, although few ice sheets may be present along the shores. Weather conditions leading up to freeze events may additionally be noted.

Ice phenology for *Lake Superior* at Bayfield, Wisconsin goes back to 1857. Data collection was started by Neil Howk and his son Forrest for his high school science project^15^. Early data regarding the opening and closing of Bayfield harbor due to ice cover were gleaned from the Bayfield County Press from 1857 until 1963 and the Island Gazette newspaper from 1964 to 1970, after which they were obtained from the Madeline Island Ferry records. After 2008, a Standard Operating procedure was established for collecting data in which Howk observed ferry operations and verified the dates with the Madeline Island Ferry Line. The dates when the ferry boat ceases and begins operations due to ice conditions were noted. Observations were made at Bayfield, Wisconsin toward the channel between Bayfield and Madeline Island. The ice-on date is the date that the last boat of the season left Bayfield harbor, whereas the ice-off date is defined as the date that the first boat of the season arrives at Bayfield harbor.

### Europe

#### Finland

*Lakes Oulujärvi*, *Kallavesi*, *Näsijärvi, Vesijärvi, Päijänne, Pielavesi, Haukivesi, Lentua, Pielinen, Visuvesi*, and *Palovesi* are all located in Finland. For these lakes (as well as for all lake ice phenology in Finland), ice-on date is defined as the final freeze-up of the lake “within sight” at the observation site (usually from a water level station) after any possible thaw events^31^. Ice-off date is recorded when the ice breaks up and is not visible from the observation site. Within sight at a small lake often means the whole lake, but it is difficult for an observer to get information about the freezing of all the parts of a large and complex lake. Ice-on is observed at four stages according to the Finnish Environment Institute’s (SYKE) observation instructions^32^, depending on the extent of the ice cover within the horizon of the observation site. The four stages of freezing are: i) freezing of the shores, ii) freezing of the bays, iii) freeze-up of the lake within sight and iv) freeze-up of the whole lake. Only a few observation sites display a clear sequence of these four stages, whereas at other sites, it is difficult to distinguish among the four. In some years, severe cold conditions cause all four stages to occur during the same night. The four stages of ice melting are: i) thawing of the shores, ii) thaw areas out of the shore, iii) ice in movement and iv) no ice within sight. If there were several records for the same year from different data sources, the earlier date was used. The duration of ice cover in days was defined as the number of days between ice-on and ice-off^31,32^.

Ice phenology records were started in the 19^th^ century to monitor hydrology, water level records, and waterways important for trading. The longest lake observations, such as in Näsijärvi and Kallavesi, began as lake harbour offices made notes of the open-water lake navigation season. Lake Oulujärvi observations from 1854–1885 were made by a local resident and they were published in a local newspaper in 1934. Since the late 1880s, lake water levels were monitored with ice phenology in many of these lakes. Typically, observations were made from the water level station towards all possible directions by a local observer who was engaged for the duty. Whole lake views were sometimes possible at some observation sites but not for the whole time series and it was not compulsory to take such observations. Observers used to take records at 8 am (the time of water level reading) but ice-on/ice-off stages were reported at any time if the observer was living nearby the station. Sometimes, the exact time is given in the paper reports by the observer, but these are not available in the database since only dates could be stored in the database. All other notes written by reporters were also kept on paper but not digitized. Remote sensing observations from MODIS and Sentinel-2 and web cameras supplement more recent observational data. However, they are not regularly used for screening because of cloudiness and the fact that many of these Finnish lakes are too small and fragmented for remote sensing. Ice data collection procedure instructions, including the different freezing and melting stages, have been written and published in Hydrological Yearbooks (1910–2010). There may have been unremarkable changes in the procedure, although there have been no changes since the 1960s according to the definitions in the yearbooks. It is estimated that the uncertainty associated with the observations was low during the 20th century when the water stations were not automated and observations were conducted daily by persons living by the station. However, over the last 5–10 years, the water level stations were automated on a wider scale, and therefore uncertainty may be larger, as not all observers live nearby, and they may visit a station only every two weeks. In some years, lakes were not visited and freezing/melting dates were estimated from ice records of other lakes. The subjective component of the observation (different observers are involved) is another factor that contributes to the uncertainty of the record^32^.

#### Hungary

The ice record for *Lake Balaton* has been on-going since 1925 from a site in Siófok, where the state of lake ice is recorded on a daily basis. These ice records were supplemented with historical ice observations from earlier studies, including “Fishing on the ice of the Lake Balaton^33^”, “The thermal regime of the Lake Balaton^34^”, and “The ice of the Lake Balaton^35^” from three different sites around the lake (Keszthely, Balatonfüred and Balatonszemes) to produce a consistent ice record from 1885 to 2016^36^. The ice-on date we report is the start date of the continuous solid ice cover period. The ice-off date is the day the ice cracks, and returns to floating ice. In years where ice phenology data were collected at multiple sites, no significant differences were observed between dates among sites^36^.

#### Iceland

*Lake Þingvallavatn*’s ice record started in 1922 by farmer Guðmann Ólafsson at Skálabrekka farm, located in the middle of the lake’s western shore. Ólafsson was keen on nature and enthusiastic about natural phenomena, thus kept diaries with detailed information on farm life, nature, and fishing logs. From 1987 and onwards, Árni B. Stefánsson, an ophthalmologist and owner of a summer house close to Skálbrekka farm, who knew Ólafsson began recording the observations. Visual observations generally took place between 10 am to 6 pm. Observations were conducted from a high location, mostly from hills on the west side of the lake, to obtain a view of the entire lake. A closer look is usually obtained with binoculars or by visiting one or two lake sites. Additional information may be collected from either residents around the lake or from lake photos available over the observation period. The ice-on date is recorded when Lake Þingvallavatn is covered throughout with land-fast ice, except for areas with spring-fed inlets mainly along the North and southwest coast (90% of the water enters the lake through subterraneous springs, 3–4 °C all year around). Ice-off date is defined as the date when the land-fast ice starts to break up resulting in open water and drifting ice-flakes. Although the procedure for collecting data has remained similar, there is large uncertainty during the period 1992–2000 due to some delays in observers’ visits. From 2001 onwards, Árni B. Stefánsson has been backing up his own observations by contacting farmers around the lake when he is away from his summer house^37^.

#### Norway

The Norwegian Water Resources and Energy Directorate (NVE) began recording ice data from *Lake Atnsjøen* in 1917 by directly observing the lake in the morning. Between 1917–1999, observers would stand at the northern shore of the lake (61.85011° N; 10.22279° E) and face towards the north. After 1999, observations were made from the northern part of the lake (61.88588° N; 10.14479° E) towards the south. Different staff of the NVE have been following the same procedure to collect this data. However, since the 1980s, the same observer has recorded the ice dates. In recent years, web-cameras and residents who live near the lake provide additional information to increase the data accuracy during critical periods. The ice-on date is recorded as when some ice first appears on the lake, whereas the ice-off date is when the lake is completely ice free. The dataset additionally contained information of daily weather conditions and water level.

*Lake Aursunden*’s ice phenology record began in 1902 by the regional hydropower company (Glommens og Laagens Brukseierforening). Between 1902–2012, the data were collected through weekly morning observations from the western shore of the lake (62.68067° N, 11.46401° E) towards the east. As the observers lived nearby, sometimes additional information was obtained in between the weekly visits particularly during critical periods of ice formation and thaw. After 2002, remote sensing including Moderate Resolution Imaging Spectroradiometer (MODIS) from the National Aeronautics and Space Administration (NASA) (up to 2015) and Sentinel-2 from the European Space Agency (ESA) (2015 onward) have been additionally used to collect ice phenology data to increase the accuracy. The ice-on date is defined when the ice is first observed, whereas the ice-off date is the date there is no ice on the lake.

The regional hydropower company (Glommens og Laagens Brukseierforening) started the lake ice phenology dataset for *Lake Tesse* in 1908 with weekly observations in the morning from the northern shore (61.80535° N; 8.94799° E) while looking southward. The ice-on date is the day when some lake ice appears for the first time and ice-off is recorded as the date that the lake is completely ice free.

*Lake Tunhovdfjorden*’s record began in 1920 by the NVE to provide information for the development of a hydropower reservoir. Weekly direct observations were made in the morning from the southern shore (60.32223° N; 8.92416° E) towards the south of the lake from 1920 to 1994. There was a large gap in the ice record during 1994–2014 after which the ice data were collected using remote sensing observations from Sentinel-2 at the lake centre (60.42573° N; 8.83726° E). The observers that lived near the lake sometimes provided additional information between weekly visits which increased the data accuracy during critical periods. The ice-on date is the day when some lake ice first appears, whereas the ice-off date is when the lake is completely free of ice.

For all of the Norwegian lakes described in this study, additional features of the ice were recorded prior to and during the time of observation including: first ice in registration site, partial freeze-up at registration site, complete freeze-up at registration site, first ice near shore, ice freeze-up near shore, partial freeze and break up if there was open water in sight of several kilometers, breaks in data record, freeze-up across the entire lake, and ice-free across the entire lake.

#### Poland

*Serwy Lake*’s ice phenology record began in 1888. The Russian Ministry of Road Information started the record to manage inland waterways and reported it annually from 1888 to 1918. After 1918, the ice record was conducted by Polish Hydrographic Service. Observations were made daily from piers about 20 m away from the southern shoreline of the lake towards the north at 06 GMT by employees of the Institute of Meteorology and Water Management - National Research Institute (IMWM – NRI) in Warsaw. Additional information such as the place of occurrence (e.g. lake shore, part of the lake, whole lake) and type of ice was also noted. Subsequently, web cameras were incorporated into the study as a complementary observation. The ice-on date is defined as the first day with ice cover in winter (but ice may melt later), when 100% of the lake area within a visible measurement sector was covered with ice. Ice-off date is defined as the last day with ice cover in winter (day before the date of disintegration of the ice cover).

#### Sweden

*Lakes Orsasjön, Näckten, Kallsjön, Storuman, Göuta, Runn, and Jukkasjärvi* are lakes located across Sweden. The systematic recording of lake ice began in the late 19th century and early 20th century, for example in 1859 for Runn, in 1870 for Orsasjön, Kallsjön and Göuta, and in 1908 for Näckten. For all of these lakes, ice-on dates are defined as the day when the main lake area is first covered by ice and the ice cover lasts for at least 3 days over at least two-thirds of the lake area. Ice-off date is defined as when the main lake area is ice free implying that some small bays may still have ice cover. If the lake freezes again, the last date when it opens is taken as the ice-off date^38^. Freeze-thaw events throughout the winter season are also recorded. The Swedish Meteorological Institute (SHMI) was founded in 1945 and started recording lake ice data for national monitoring using a Standard Operating Program established in the national ice monitoring program. The institute also collected older ice observations and homogenized them. They further used visual observations (including daily observations for larger lakes) as the primary means to collect data which were supplemented with satellite imagery in the last few years. Although the procedure for collecting data was consistently used throughout the study and the SHMI’s staff was highly experienced, there may be limited uncertainty associated with the variability in personal judgements and observations.

#### Switzerland

Ice coverage information from ten lakes located on the Swiss plateau, an area in Switzerland with altitudes between 400–700 m, is included in the database. As the average winter air temperatures in this area of Switzerland are slightly above the freezing point, these relatively deep lakes no longer freeze each winter. For these lakes, a common definition was used for ice-on and ice-off dates. The ice-on date is defined when the lake is completely or almost completely (>90%) covered with ice for more than one day. On the other hand, the ice-off date is defined as the date when more than 10% of the lake has open water (this implies that less than 90% of the lake is still frozen). In years when the historical information is from newspapers, there may be uncertainty in the dataset of a few days. It is possible that freezing events were not reported by newspapers, and would be missed. We believe that this risk is in general small, as the freezing of larger lakes did not occur each winter, and had economical (e.g., fishery), and socio-cultural (e.g, ice skating) implications. Visual observations might be especially affected in case of impaired visibility (e.g., fog). For some of the lakes systematic registrations were made, which are expected to be associated with less uncertainty (Hendricks Franssen and Scherrer, 2008).

*Lake Aegeri*’s ice phenology record began in 1914. Observers examined the lake from different locations around the shore. The information was gathered from several sources: i) the newspaper Zuger Zeitung (1914 - present), ii) systematic handwritten chronicle (1914–1964), iii) Livingstone’s scientific publication (1964–1988)^39^, iv) lake police’s systematic registration and some non-systematic registration of fishermen (1981–2006), and v) webcams and restaurant owner on the lake shore (2007 - present).

Freeze and thaw events in *Lake Baldegg* are gathered from a local newspaper, diaries, and personal communication. In 1891, Naturforschenden Gesellschaft Luzern started the record and continued for the first ten years for scientific investigation purposes. After 1901, information was collected via newspapers (specifically Luzerner Zeitung) up to 1955. Family Hofer, in addition to fishermen on Lake Baldegg and Lake Sempach continued the record based on visual observations up to recently. Ice is observed on the lake at various locations visually.

*Lake Biel*’s ice record was started by the fishermen Arnold Martin, out of his curiosity and for fishing purposes in 1923. From 1923 to 2006, ice phenology information was observed and systematically recorded each year into the family’s diary. Arnold Martin and family would observe the lake mostly in the morning in the southern direction from the shore or on boats while on the lake. Measurements of ice depth and percent ice coverage were also noted. Since 2007, data have been obtained unsystematically from newspapers with additional information provided by webcams. Limited visibility can lead to overestimation of ice coverage.

Local newspapers were used to gather early ice records for *Lake Greifen* from 1901 to 1948. Between 1949 and 1980, data were gathered from von Eugen and Orn^40^. From 1963 onwards, data have also been obtained from the lake security service’s observations distributed around the lake, in addition to webcams, and information from boats used for daily passenger traffic over the lake. Webcams observe the lake from the eastside opposite of Maur in the northwest and south directions. Boats, on the other hand, collect data from Maur to Uster (middle of lake). Observations are done mostly in the morning. The uncertainty of the data before 1949 is larger than data more recently.

*Lake Hallwil*’s ice phenology records began in 1901 and data were collected from the newspaper Luzerner Zeitung (1901–1954), the boat designer Urs Merz (1955–2010), and a webcam combined with social media afterwards. Web cameras provided observations for ice coverage information from high locations due east in the morning. Urs Merz made many of the observations for most of the time series before he passed away. Taking into consideration the diverse information sources from the observers and other villagers along the lake, the data is likely reliable.

*Lake Murten*’s ice record began in 1901. Data were first obtained from the local newspaper Murtenbieter for the first 70 years (to 1970), after which it was based on observations of fishermen until 2006. After 2006, ice data have been obtained from webcam images and information from a boat service across the lake. The lake is observed in the northwest direction over the lake. Additionally, for Lake Murten, newspapers and fishermen reported information of freeze and thaw events, although the fishermen did not systematically record freeze-thaw events after 1971.

*Lake Pfäffikon*’s ice record began in 1922. The earliest data were collected from newspapers. From 1942 to 1999, ice phenology information was supplemented with visual observations from Anton Hiestand (1942–1999) and von Eugen & Örn^40^ (data for 1948–1980). Observations were usually performed in the morning at multiple locations while observers were looking over the lake. Anton Hiestand and von Eugen & Örn^40^ also provided two time series of systematic registration for freeze-thaw events which were also reported in newspapers. In addition, webcam images (since 2003) and social media have provided additional ice records.

The ice record in *Lake Sarnen* began in 1901. Data were mostly obtained from the newspaper Obwaldner Volksfreund. In addition, the lake police and the fisher family Hofer have been providing scattered ice information based on their observations since 1971, and webcam images have been used since 2007. Observations were made due southwest and north while observers took notes of ice phenology information including ice depth and ice coverage. In the period between 1947 and 1954, more information was provided from personal diaries and systematic registration. Only from 1947 to 1954 freezing events were registered systematically by observers at site, while for other periods the information on ice cover was only written down in case of freezing events of interest.

Freeze and thaw events in *Lake Sempach* have been recorded since 1891. Data from 1891 to 1900 were recorded by Naturforschenden Gesellschaft Luzern, who started the scientific record. Ice phenology dates that occurred between 1901 and 1955 were noted in the Luzerner Zeitung newspaper. From 1955 onward, they are recorded in the fishermen family Hofer’s diary. The family would stand at the northwestern part of the lake in Oberkirch and look over the lake in the southeastern direction to obtain information. In addition, the degree to which the lake is frozen is noted.

*Lower Lake Constance (Untersee)*’s ice phenology record started in 1887 by the Iceclub Steckborn to manage outdoor ice-skating activities. The ice club also kept the record of most freeze and thaw events, in addition to ice depth measurements. After 1955 up to 2012, ice phenology records were obtained from newspapers such as Newspaper Bote vom Untersee and Rhein to complete the data set. Iceclub Steckborn continues to perform observations from 1955 until present, but with less systematic registration in diaries. Webcams have been incorporated into the study since 2006. Webcams were set up along the southern lake border in Steckborn, Switzerland and directed due north to obtain images of the lake in the morning. Other points of the southern border of Lower Lake Constance such as Ermatingen are also observation sites. Social media is an additional means of collecting data. It is believed that the ice club observations are very precise.

*Lej da San Murezzan* is located in an alpine valley and not on the Swiss plateau. The ice dataset for Lej da San Murezzan was started in 1831. Both the ice-on and ice-off data were collected based on visual observations. The methodology for collecting data in years prior to 2016 was unavailable. However, after 2016, observations were conducted on an hourly basis with assistance of two webcams at the north shore (St Moritz) that provide images toward the south and east directions for most of the lake. Additional information was obtained from water temperature measurements performed at the center of the lake. Since temperature-based estimation is an indirect method, there is an uncertainty owing to the lag time between webcam and temperature monitoring.

### Asia

#### Japan

*Lake Suwa*’s ice-on record was officially started in 1443 by the The Ohori family of the Suwa Grand Shrine. The ice records were collected primarily for religious purposes based on the worship of nature. The appearance of the sinusoidal ice ridge, the *omiwatari*, is traditionally interpreted by the shintoist tradition as the mark of a god who walked across the frozen lake. The direction of the cracks of the omiwatari was used as a signal to forecast the agricultural harvest for the coming summer. Observers stand at the shoreline of the lake and look towards the lake centre. The observation is often conducted at a single point view at the mouth of the Funato River, but sometimes the observers may confirm the ice-on or *omiwatari* from multiple sites, especially since the advent of vehicles which allowed observations at multiple sites. During the era of the current Shinto priest, Mr. Kiyoshi Miyasaka, observations were conducted by the Chief Priest (Guji in Japanese) and some parishioners (Ujiko in Japanese) who live in Takashima (later Kowata) village at 6:30 AM every day from the start of January to the start of February. After 1897, people from Yatsurugi Shrine and Suwa Meteorological Observatory were trained to observe ice data of the lake. The ice-on date is recorded when the lake is completely covered with ice. For Lake Suwa, the date of the appearance of a sinusoidal ice ridge also called the “*omiwatari*” in Japanese is further noted. The data collection procedures may have slightly changed over the time-series because the principal reason for the observations was to forecast the agricultural harvest in the coming summer based on the direction of the *omiwatari*, so the ice-on dates did not need scientific accuracy. No one knows exactly how the procedures have changed. The information was reported annually, although ice-on dates are missing and uncertain during the middle of the record. For example, the years between 1682 and 1896 are uncertain because of changes in the calendar from lunar to Gregorian and confusion between recording the ice-on date, *omiwatari* date, or the *omiwatari* ceremony date^2^.

#### Russia

*Lake Baikal*’s ice phenology record began in 1896 in Listvyanka (located at the northwest shore of south Baikal) to control the goods transported across the lake between Russia and China. Data in the years prior to 1950 were collected from different historical records by head of department of Hydrology and Hydrophysics (1960–1971), Limnological Institute, Siberian Branch of Russian Academy of Sciences, Vladimir Sokolnikov. After 1950, ice data have been attained via visual observations by Anna Ponomareva, Matrena Alsaeva, and Ekaterina Fomina (1950–1990); in addition to Andry Zhdanov, Rita Zhdanova and Elena Salva (1991 - present). Observers would conduct observations during the afternoon at the pier of boats 30 m away from the shore in Listvyanka while facing south and looking from east to west. Percentages of open water and ice coverage are noted during the observation progress. The ice-on date is recorded when lake Baikal is completely covered with ice. When more than one percent of the lake is open water, the date is recorded as the ice-off date. Freeze/thaw events as well as a description of freezing and thawing processes are additionally documented at various locations across the lake.

## Data Records

All data is open access and can be found on Figshare^41^. The datasets were split into three main files that include ice-on and ice-off dates (PhenologyData.csv), the physical characteristics of each lake including the geospatial coordinates (LakeCharacteristics.csv), and corresponding metadata for each lake, including the number of missing years and definitions of ice-on and ice-off (Definitions.csv). All datasets can be connected by the unique lake name (column name = lake) that is present in each. For those interested in examining patterns in ice phenology among lakes, the PhenologyData.csv would be all that is required. We also recommend downloading additional ice phenology data for hundreds of lakes and rivers around the Northern Hemisphere from the Lake and River Ice Phenology (LIAG) dataset stored at the National Snow and Ice Data Centre (NSIDC^42^) or within Canada specifically from the Canadian Ice Database housed at the Polar Data Catalogue^43^. Some of the lakes provided in this study are a subset of those found within LIAG, although unfortunately detailed data descriptors had not been previously collected. For others interested in using the data in combination with remotely sensed data or downscaled climate models, we recommend joining the LakeCharacteristics.csv to the PhenologyData.csv and the Definitions.csv by the “lake” column. Potential resources that could be relevant for testing changing patterns in lake ice include the HydroLAKES database for geomorphic data^44^, Moderate Resolution Imaging Spectroradiometer (MODIS) for spectral bands to be used in remote sensing, or Climatic Research Unit of East Anglia for monthly climate values^45^. We also recommend WorldClim for historical and future climate patterns under multiple general circulation models and four shared socio-economic pathways^46^.

Although we could not precisely quantify uncertainty in ice-on and ice-off dates for these 100+ year long datasets, the uncertainty for many of the lakes may be in the order of magnitude of one day to several days. General factors contributing to uncertainty include: variability in personal judgement between lake ice observers, low visibility, or a lake freezing over the weekend or holidays. For example, in Wilson Lake, different observers sometimes report dates that are one day apart for the same year. Low visibility, including fog, incomplete views of lakes, and nightfall, such that a lake freezes overnight and has melted by the morning, can contribute additional uncertainty in ice-on and ice-off dates.

## Technical Validation

We conducted quality assurance and quality control on the database to ensure each observation was accurate. The data were reviewed by multiple members of the team using similar methods to check for discrepancies. There were three components that required specific attention during review including: 1) lake names, 2) lake physical characteristics, 3) dates for ice-on or ice-off.

Lake names were reviewed for consistency among observation records. In many instances, lakes had inconsistent names due to differences in locations surveyed, anglicization of lake names, or local synonyms. For instance, Lake Baikal was occasionally reported as Listvyanka, the town where the observations of the lake was reported. In another instance, Cobbossee Lake is often used for Cobbosseecontee Lake, presumably a local colloquialization of the name. We reviewed each of the lake names and created consistent labelling with each lake name being all lower case and multiple words separated with an underscore. For each lake name, we did not include any mention of the word “Lake” (except in the case of “Haystack Bay, Lake of Bays”). Accented or non-ASCII characters were replaced with non-accented, similar alphabetic equivalents. We included within our database a column that represents identically the italicized names of the lakes reported within this manuscript (LakeCharacteristics.csv).

We obtained geospatial coordinates from the Lake Ice Analysis Group (LIAG), part of the National Snow and Ice Data Center (NSIDC). There were twelve lakes that were not matched in the NSIDC database and were instead collected by contacting the original data providers. We reviewed these coordinates to ensure each occurred in the identified lake. In a few instances, we adjusted the coordinates of lakes that were slightly outside of the water to be located near the center of the lake. These discrepancies would occur because latitude and longitude from NSIDC were only reported to two decimal places, which often lacks the precision to accurately identify a point within the lake. Some observations had different geospatial coordinates but were located within the same lake. For consistency, we use the same latitude and longitude for all observations within the same lake. We collated characteristics and morphology of the lakes including elevation, surface area, mean lake depth, maximum lake depth, and shoreline length. We also provide columns for the data source that was used to obtain these data. For instance, in many lakes, we were able to acquire this information from the LIAG database, but in other instances, we used very specific resources such as the Norwegian Water Resources and Energy Directorate for the Norwegian Lakes. We were not able to obtain lake characteristics and morphology for all variables and all lakes.

The dates of ice-on and ice-off were reviewed for consistencies among lakes and anomalies among years. The first verification step was to confirm the correct year, as the 2019–2020 ice season, for example, would alternately be reported as 2019 or 2020; the latter of these, sensibly, was used in cases where only ice-out is recorded. All dates were revised to follow the format of year-month-day with year being in a four-digit format (i.e. YYYY-MM-DD). When no data were available, a blank was reported. Most of the database provides information for the longest continuous ice presence, but in some cases there were intermittent freeze-thaw events. We provide a column specifying the freeze event that occurred when multiple freeze thaws would occur. Each additional freeze-thaw event would be labelled sequentially (i.e., froze_1, froze_2,… froze_i). This formatting allows for recording of a near infinite number of freeze-thaw events. However, lakes rarely had more than three ice on-off dates. For a given season, ice-on has to occur before an ice-off event. We reviewed observations where ice-off dates were reported to occur before ice-on dates. There is typically a linear relationship between ice-on and ice-off dates. Therefore, to check for anomalies in dates, we conducted regressions between ice-on and ice-off day-of-year. Dates that departed considerably from the linear pattern were reviewed to ensure accuracy. In some instances, the original observer or data collector from the lake was contacted to verify the date. We also checked the difference of the minimum and maximum date relative to the next closest value within the timeseries to determine if there were transcription errors. If the data provider included a “duration” column we added it to our dataset, but we found that these values were not consistent with the difference between ice-off date and ice-on date. Two of the most common reasons why the duration column had inconsistent estimates was because 1) some providers only subtracted ice-on date from ice-off date, while others added 1 to make it an inclusive duration, and 2) in years with multiple freeze-thaw events, some providers took the difference from the first ice-on and last ice-off, but others summed only the days when the lake was ice covered (i.e. only counted the ice frozen days).

## Data Availability

We provide code in an open access repository that will assist in reviewing the database and examining patterns (https://github.com/afilazzola/IcePhenologyDatabase). Within the repository, we provide the Python code used for the creation of the dataset, quality assurance, and quality control. We included a set of visualization options for technical validation in Python. All Python code was conducted in Python Version 3.8.1 (http://www.python.org) using numpy^47^, pandas^48^, textract libraries. We also provide R code that was used as a second reviewer for technical validation. The code in the qaqc.r file was used to convert the dataset into “long” format where there is only one column each for ice-on and ice-off date. The same file may also be used by future users for a quick visualization of patterns within the lake ice database. All R code was conducted in R version 3.5.1^49^ using the tidyr, dplyr, ggplot^50^, and broom packages. All code is freely available under the Massachusetts Institute of Technology license.
