# Author Correction: Long-term ice phenology records spanning up to 578 years for 78 lakes around the Northern Hemisphere

**DOI:** 10.1038/s41597-022-01531-y

**Published:** 2022-07-18

**Authors:** Sapna Sharma, Alessandro Filazzola, Thi Nguyen, M. Arshad Imrit, Kevin Blagrave, Damien Bouffard, Julia Daly, Harley Feldman, Natalie Feldsine, Harrie-Jan Hendricks-Franssen, Nikolay Granin, Richard Hecock, Jan Henning L’Abée-Lund, Ed Hopkins, Neil Howk, Michael Iacono, Lesley B. Knoll, Johanna Korhonen, Hilmar J. Malmquist, Włodzimierz Marszelewski, Shin-Ichiro S. Matsuzaki, Yuichi Miyabara, Kiyoshi Miyasaka, Alexander Mills, Lolita Olson, Theodore W. Peters, David C. Richardson, Dale M. Robertson, Lars Rudstam, Danielle Wain, Holly Waterfield, Gesa A. Weyhenmeyer, Brendan Wiltse, Huaxia Yao, Andry Zhdanov, John J. Magnuson

**Affiliations:** 1 Department of Biology, York University, 4700 Keele Street, Toronto, Ontario M3J 1P3 Canada; 2 Eawag, Swiss Federal Institute of Aquatic Science and Technology, Surface Waters - Research and Management, Kastanienbaum, 6047 Switzerland; 3 Department of Geology, University of Maine- Farmington, 173 High Street, Farmington, Maine 04938 USA; 4 Chanhassen, MN 55317 USA; 5 Mohonk Preserve, New Paltz, NY 12561 USA; 6 Agrosphere (IBG-3), Forschungszentrum Julich GmbH, Julich, Germany; 7 Department of Hydrology and Hydrophysics, Limnological Institute, Siberian Branch of Russian Academy of Sciences, Irkutsk, Russia; 8 Lake Detroiters Association, Detroit Lakes, MN 56501 USA; 9 Norwegian Water Resources and Energy Directorate, Box 5091, Majorstuen, N-0301 Oslo Norway; 10 Wisconsin State Climatology Office, University of Wisconsin-Madison, Madison, Wisconsin USA; 11 Bayfield, WI 54814 USA; 12 Blue Hill Observatory Science Center, PO Box 187, Readville, MA 02137 USA; 13 Itasca Biological Station and Laboratories, University of Minnesota Twin Cities, 28131 University Circle, Lake Itasca, Minnesota 56470 USA; 14 Finnish Environment Institute SYKE, Freshwater Centre, Latokartanonkaari 11, 00790 Helsinki, Finland; 15 Icelandic Museum of Natural History, Suðurlandsbraut 24, 108 Reykjavík, Iceland; 16 Nicolaus Copernicus University in Toruń, Faculty of Earth Sciences and Spatial Management, Lwowska 1, 87-100 Toruń, Poland; 17 Biodiversity Division, National Institute for Environmental Studies, Onogawa 16-2, Tsukuba, Ibaraki 305-8506 Japan; 18 Shinshu University, Kogandori 5-2-4, Suwa, Nagano, 392-0027 Japan; 19 Yatsurugi Shrine, Suwa, Nagano, 392-0024 Japan; 20 Washburn County, County Clerk’s Office, 10 4th Avenue, PO Box 639, Shell Lake, WI 54871 USA; 21 Geneva Lake Environmental Agency, George Williams College, 350 Constance Blvd., Williams Bay, WI 53191 USA; 22 Biology Department, State University of New York at New Paltz, 1 Hawk Drive, New Paltz, NY 12561 USA; 23 U.S. Geological Survey, Upper Midwest Water Science Center, Madison, WI 53726 USA; 24 Cornell Biological Field Station, Department of Natural Resources and the Environment, Cornell University, Bridgeport, NY 13030 USA; 25 7 Lakes Alliance, 137 Main Street, Belgrade Lakes, ME 04918 USA; 26 Biological Field Station, State University of New York College at Oneonta, 5838 State Hwy 80, Cooperstown, NY 13320 USA; 27 Dept. of Ecology and Genetics/Limnology, Uppsala University, 75236 Uppsala, Sweden; 28 Adirondack Watershed Institute, Paul Smith’s College, 7777 NY-30, Paul Smiths, NY 12970 USA; 29 Dorset Environmental Science Centre, Ontario Ministry of Environment, Conservation and Parks, 1026 Bellwood Acres Road, Dorset, Ontario P0A1E0 Canada; 30 Center for Limnology, University of Wisconsin-Madison, 680 North Park Street, Madison, Wisconsin 53706 USA; 31 Present Address: Basaltveien 39, N-1359 Eiksmarka, Norway

**Keywords:** Limnology, Cryospheric science, Limnology, Cryospheric science

Correction to: *Scientific Data* 10.1038/s41597-022-01391-6, published online 16 June 2022

In this article the author name Natalie Feldsine was incorrectly written as Natalie Felsine. The original article has been corrected.

